# Vision, cognition, and walking stability in young adults

**DOI:** 10.1038/s41598-021-04540-w

**Published:** 2022-01-11

**Authors:** Yogev Koren, Rotem Mairon, Ilay Sofer, Yisrael Parmet, Ohad Ben-Shahar, Simona Bar-Haim

**Affiliations:** 1 Physical Therapy Department, Ben-Gurion University of the Negev, Be’er-Sheva, Israel; 2 Computer Science Department, Ben-Gurion University of the Negev, Be’er Sheva, Israel; 3 Industrial Engineering and Management Department, Ben-Gurion University of the Negev, Be’er Sheva, Israel; 4 Zlotowski Center for Neuroscience, Ben-Gurion University of the Negev, Be’er Sheva, Israel; 5 Negev Lab, Translational Neurorehabilitation Laboratory, Adi-Negev, Nahalat Eran, Ofakim, Israel

**Keywords:** Attention, Cognitive control, Perception, Motor control

## Abstract

Downward gazing is often observed when walking requires guidance. This gaze behavior is thought to promote walking stability through anticipatory stepping control. This study is part of an ongoing effort to investigate whether downward gazing also serves to enhance postural control, which can promote walking stability through a feedback/reactive mechanism. Since gaze behavior alone gives no indication as to what information is gathered and the functions it serves, we aimed to investigate the cognitive demands associated with downward gazing, as they are likely to differ between anticipatory and feedback use of visual input. To do so, we used a novel methodology to compromise walking stability in a manner that could not be resolved through modulation of stepping. Then, using interference methodology and neuroimaging, we tested for (1) interference related to dual tasking, and (2) changes in prefrontal activity. The novel methodology resulted in an increase in the time spent looking at the walking surface. Further, while some dual-task interference was observed, indicating that this gaze behavior is cognitively demanding, several gaze parameters pertaining to downward gazing and prefrontal activity correlated. These correlations revealed that a greater tendency to gaze onto the walking surface was associated with lower PFC activity, as is expected when sensory information is used through highly automatic, and useful, neural circuitry. These results, while not conclusive, do suggest that gazing onto the walking surface can be used for purposes other than anticipatory stepping control, bearing important motor-control and clinical implications.

## Introduction

Walking is a complex human behavior controlled by spinal and supra-spinal structures^1^. While the control of healthy steady-state walking is thought to be highly automatic^2^, it does require attention and other cognitive resources^3^ involving the cerebral cortex^4^. One area of particular interest is the prefrontal cortex (PFC), which has been studied extensively in the context of human locomotion^5,6^. This area is thought to play a central role in “top-down” regulation^7^, which is required for voluntary behavior, namely, when translating internal goals into actions. The activity of this area has been reported to increase when intentional modulation of gait is required^8–12^ and is thought to reflect a shift from automatic to conscious control of walking^2^.

In visually guided behavior, eye movement is goal-directed, representing the shift of attention from one area of interest to another. In that context, a tight coupling between gaze behavior and stepping has been reported in many situations where visual information serves to modulate gait^13^. Specifically, downward gazing has been repeatedly observed when individuals are negotiating obstacles and/or when they are required to locate the next foothold/s position (e.g.,^14–16^). Walking gaze behavior has been suggested to provide information used, primarily, in an anticipatory manner to plan future actions/steps^17^, but also to guide ongoing ones^18^. This indicates that attention and possibly other cognitive resources are required to acquire, process, and use visual information to control gait (i.e., plan or guide subsequent or current step/s). This perspective is supported by reports on cognitive-visual interference^19–21^; a limited number of reports indicate that gaze behavior associated with conscious stepping control is susceptible to cognitive interference^19–23^. Importantly, an increase in time spent looking at task-irrelevant areas was observed under cognitive load^20,21^, as if participants were trying to “disengage” from task-relevant visual information. Therefore, it only stands to reason that gaze behavior dedicated to anticipatory control of stepping is cognitively demanding, possibly involving the PFC.

Much like gait, postural control is also cognitively demanding^24^, involving the PFC^25^. These demands have been reported to increase when postural demands increase^26^. An extensive body of evidence indicates that visual information is very useful for postural control^27–30^. However, as opposed to visual information used in an anticipatory manner (to modulate stepping) to maintain walking stability, visual information used for postural control supports stability through a feedback/reactive control mechanism. This is true for both standing^27^ and walking^31^. For standing, such control has been suggested to promote automaticity^32^ and shown to reduce PFC activity^33^. For steady-state walking, increased PFC activity was observed when visual input was reduced, due to dim lighting, although anticipatory control of stepping was redundant^11^. This observed effect is similar to those observed for deficits in other sensory modalities, which largely support walking through feedback control loops (reviewed in^2^) and thus indicate that when sensory information required to control walking is inadequate or absent, automaticity decreases.

Recently, Koren et al.^34^ reported that gazing down, just a few steps ahead, increases the postural steadiness of standing and walking healthy adults. Further, these authors also reported that under threat to walking stability, healthy adults increased the time spent gazing down, even though anticipatory control of stepping could not resolve the stability problem in their paradigm^35^. Taken together, these results may indicate that downward gazing behavior can be and is used to support walking stability not only through anticipatory stepping control, but also through feedback postural control. While this conclusion is quite reasonable, it is also possible that, under threat to stability, humans shift from automatic to conscious control of stepping^36^, and the mere fact that the authors’ participants gazed down does not necessarily imply what function/s this gaze behavior serves.

To address this problem, in this study we explore the cognitive demands and PFC activity associated with walking instability. In particular, we aimed to investigate the association of these cognitive demands with downward gazing behavior, under the same conditions reported by Koren et al.^35^; the underlying assumption was that the cognitive demands should be different if visual information is used for anticipatory stepping control, as opposed to feedback/reactive postural control. To this end, we used interference methodology during unperturbed and perturbed walking conditions, and monitored gaze behavior and PFC activity to establish (a) whether or not downward gazing is susceptible to cognitive interference, and (b) to establish whether downward gazing is associated with an increase or decrease in PFC activity. The results of this investigation are discussed in the context of the mechanism/s through which visual information can support walking stability.

## Methods

The present study shares some experimental equipment and measurements from an earlier report^35^ (currently under review), but significantly extends the experimental protocols and analyses, including a large volume of new unpublished data. Most of the details pertaining to the methods used in this experiment were previously reported^35,37^ and are found in the *Supplementary Materials Section* of this report.

### Experimental model

**Figure 1:**
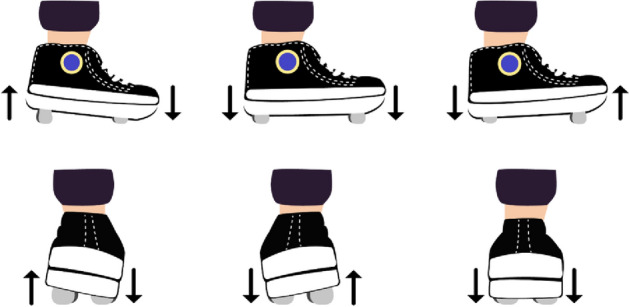
The RS has four pistons underneath each shoe. By changing their lengths, the pistons create a single plane that may be parallel or oblique to the sole’s plane. These changes take place during the swing phase, of each step, to provide a new and unpredictable support surface that is perceived only during the stance phase.

Briefly, in this experiment we monitored the gaze behavior and prefrontal activity of participants walking with their own shoes and with the Re-Step system (RS). The RS is a shoe-like device (see Fig. 1), originally developed for training and gait rehabilitation of people with brain damage^38^. The RS has four pistons on its sole that align to create a single plane. The length of these pistons may change, during the swing phase of walking, to create a plane oblique to that of the sole. These changes are perceived, during the stance phase of walking, as walking up or down hill with a leftward or rightward slope (up to 6°). Each shoe is controlled independently using a chaotic algorithm to determine the direction and magnitude of inclination, as well as the vertical displacement (i.e., elevation of up to 18 mm). This control strategy was designed to prevent prediction from one foot to the other and learning over time. Moreover, since these changes cannot be perceived visually, using visual information in an anticipatory manner to resolve the induced instability is unlikely. Namely, gazing down to acquire information required to plan future steps is futile and therefore unlikely. In this study, changes occurred in every step, and the magnitude of perturbations was allowed to reach maximal values (i.e., up to 6° of inclination and 18 mm of elevation).

### Testing procedure

For testing, participants were instructed to walk at a comfortable pace, in a well-lit hallway, along a 20-m-long course with their own shoes and with the RS. To familiarize participants with the RS system, they walked the entire course six times before testing commenced. Participants performed 12 consecutive walks in each shoe type (i.e., two blocks consisting of 12 walks each). Before each walk, participants stood silently for 25 s, after which they were presented with a tablet displaying either a simple countdown from F to A or a random sequence of six digits, both at a 1-Hz frequency. Following the countdown/sequence, a “GO” cue was presented to indicate that the participant should start walking. Participants were instructed to memorize the random sequence, but to ignore the countdown. At the end of walks preceded with a random sequence, participants reported what they recalled and their recollection was recorded. In our instructions, we emphasized that both recall of the digits and their order were important. The order of blocks and the tasks within blocks were randomly assigned to each participant.

### Participants

Participants were healthy young adults, reporting no neurological, orthopedic, or other illness affecting gait. The experiment was approved, beforehand, by the Local Committee for Research Involving Humans at the Faculty of Health Science, Ben-Gurion University (30–2017) and conformed to the standards set by the Declaration of Helsinki. All participants provided written informed consent prior to their testing and received a monetary reward for their time (the equivalence of 11$ in local currency).

### Devices

During the entire experiment, both gaze position and PFC hemoglobin concentration were monitored. Gaze position was monitored, using a binocular eye-tracking glasses (ETG, SensoMotoric Instruments, Teltow, Germany), at a 60-Hz sampling rate. For data collection, we used the software provided by the manufacturer (BeGaze, version 3.5), which references gaze positions to a scenery video from a front-mounted camera.

Changes in hemoglobin concentration were monitored using a wireless, continuous-wave fNIRS device (*PortaLight*, Artinis, The Netherlands). In this study, two devices were used to monitor the left and right hemispheres in the frontopolar area. Sample frequency was set to 10 Hz and was continuous throughout the experiment. Raw intensities were collected using the software provided by the manufacturer (Oxysoft, version 3.0.53).

Processing of the gaze data was performed automatically, using a dedicated MATLAB (version R2016b. Natick, Massachusetts: The MathWorks Inc.) script, in a frame-by-frame manner. Specifically, based on the geometry of the corridor, the script computes the position of the vanishing point in each frame of the scenery camera. Gaze coordinates were then referenced to this point, and the vertical and horizontal distances were determined (i.e., distance between Y- and X-axis values, respectively, of the gaze coordinate and those values of the vanishing point). For simplicity, values of the vertical distance were inversed such that small values represented a downward-directed gaze and large values represented a gaze directed straight ahead (closer to the vanishing point). For the horizontal gaze position, negative values represented positions to the left of the vanishing point and positive values represented those to the right. All values were reported as a percentage of the frame size. In addition, we also computed the time spent looking onto the walking surface (denoted as OnPath and given in percent from the total walking time) and the gaze look-ahead distance in those instances. Since gaze-position data is highly variable, we used the median value as the center measure.

To further explore gaze behavior (as a post-hoc analysis), we divided the look-ahead distance into three distance categories: up to 3 m ahead (Short), 3–10 m ahead (Mid), and more than 10 m ahead (Long). For each trial/walk we counted the number of frames in which gaze distance was within a certain category. This number was translated into a percentage of the total walking time. The length of the Short distance category was selected because it contains the range of gaze distances we had previously investigated and found to increase postural steadiness^34^. The length of the Long distance category was selected because we assumed that, under the current experimental conditions, visual cues contained in the walking surface would appear blurry and indistinguishable from one another, and therefore could not be used for postural control. As for the Mid distance category, this range of distances may or may not be used for postural control.

For preprocessing PFC hemoglobin concentration data, we used the same methods previously described by Koren et al.^37^ including data quality assessment, using *Homer2* (version 2.1), a free MATLAB-based toolbox^39^. Following the preprocessing stage, the walking period of each trial was normalized to 200 data points using spline interpolation to overcome differences in trials’ durations. Mean value was then calculated for each trial, hemisphere, and channel (the device uses three channels with different source-detector distances—30, 35, and 40 mm) to represent change in PFC activity. Since the devices used can measure only relative hemoglobin concentrations (i.e., change from some baseline), these values of oxy- and deoxy-hemoglobin were extracted from the time series and subtracted from the preceding rest period (i.e., the last 5 s of rest, just before the tablet was presented), which served as baseline activity. Typically, an increase in oxy-hemoglobin (HBO) and a decrease in deoxy-hemoglobin (HHB) concentrations are observed in active brain areas^40^.

To quantify the performance of the memory task, we used two parameters: the number of correct digits and the number of correct positions recalled (i.e., order). To define “correct position” we used two methods: (1) the number of digits reported in their correct position in order, and (2) the longest correct sequence. This second method was used because in several cases participants forgot the first digit but reported the correct sequence of the rest of the digits. Using the first method to score this performance would have resulted in a score of zero, which would have been non-representative of what was a fair performance of the task. To score the number of correct positions, we used the method producing the higher score.

### Statistical analysis

For statistical analysis, we used linear mixed-effect models with participants as the *random* effect, and task type, shoe type and their interaction as the *fixed* effects. For correlation analysis we used shoe type and the appropriate gaze variable as *fixed* effects. All tests were performed using SPSS (Version 26.0. Armonk, NY: IBM Corp) and in all cases, significance level was set to α < 0.05. For post-hoc testing, sequential Bonferroni was used to correct for multiple comparison, when appropriate. In several cases a logarithmic transformation was used (i.e., log_e_(value + 1)) to overcome normal-distribution deviations and the value zero. Transformed values are indicated as such in the text.

To test the effect of shoe type on performance in the cognitive task we used a binary logistic distribution (i.e., logistic regression). We did so because in most walks, the participants’ scores were perfect, and in most imperfect cases, only one or two points were detracted from the full score. Thus, we transformed these scores into binary scores, namely, “Perfect” or “Imperfect”.

## Results

Fifteen young adults took part in this experiment. In two cases the fNIRS data were of poor quality and could not be analyzed, and in one case gaze behavior of the participant was so erratic it could not be analyzed. All data of these participants were excluded from analysis. In one other case, the RS malfunctioned in the middle of the experiment and the missing data were replaced with the average of the participant in each condition. Overall, data from twelve participants, who had had data from both devices, were analyzed and are reported. Descriptive statistics of these participants are presented in Table 1. All participants reported to be healthy and to have normal or corrected-to-normal visual acuity.

**Table 1:** Descriptive statistics of participants.

	Mean	Range
Age (years)	24.8	20–30
Weight (kg)	73	47–103
Height (cm)	170	154–190
Gender (male/female)	7/5	

**Figure 2:**
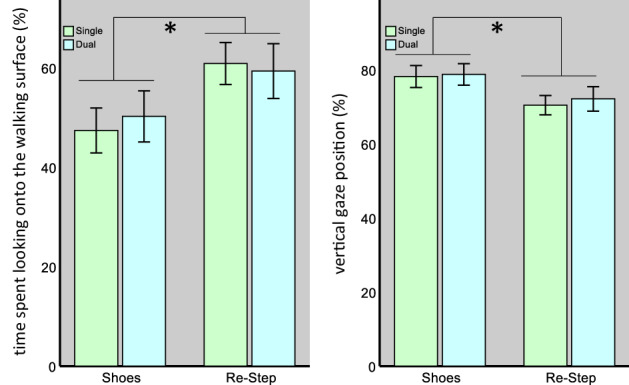
The effects of the shoe type and task type on the percentage of time spent looking at the future path (left) and the vertical gaze position (right). [*] indicates a significant main effect for the shoe type. Bars represent mean values, and error bars the 95% CI of the mean.

**Figure 3:**
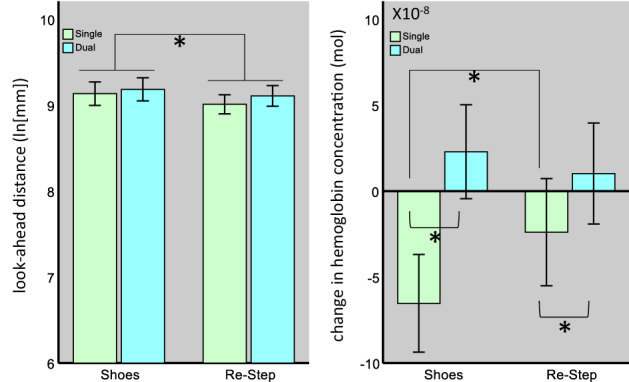
The effects of the shoe type and task type on gaze distance (left) and on PFC activity (right). For gaze distance, we found a significant main effect for the shoe type. For PFC activity, we found a significant main effect for the task type and a significant interaction term, indicating that, for both shoe types, values increased from the single- to the dual-task condition, but the difference between shoe types was significant only during single-task walking. [*] indicates a significant effect. Bars represent mean values, and error bars the 95% CI of the mean.

*Gaze behavior* (Figs. 2 and 3)- the effect of shoe-type on gaze behavior is elsewhere reported^35^, and is briefly presented below as part of the statistical models used for analysis.

First, we tested the amount of time participants spent looking onto the walking surface (OnPath). The results of this model revealed a significant main effect of the shoe type (F_1,284_ = 40.4, *p* < 0.001), but not task type (F_1,284_ = 0.14, *p* = 0.71). Their interaction was also non-significant (F_1,284_ = 1.5, p = 0.22). The main effect of the shoe type indicated a mean difference of 11.29% ± 1.67SE (*p* = 8.2 × 10^–10^) between the RS (60.1% ± 4.45SE) and the shoe (48.8% ± 4.45SE) conditions.

Next, we tested how the vertical gaze position was affected by the different conditions. The results of this model also revealed a significant main effect of the shoe type (F_1,284_ = 57.1, *p* < 0.001), but not task type (F_1,284_ = 1.4, p = 0.24). Their interaction was also non-significant (F_1,284_ = 0.36, *p* = 0.55). The main effect of the shoe type indicated a mean difference of 7.16% ± 0.95SE (*p* = 5.67 × 10^–13^) between the shoe (78.5% ± 2.98SE) and the RS (71.3% ± 2.98SE) conditions.

Testing of the horizontal gaze position revealed no main effect for the shoe type (F_1,284_ = 0.23, *p* = 0.64) or the task type (F_1,284_ = 0.07, *p* = 0.79), and their interaction was also non-significant (F_1,284_ = 1.33, *p* = 0.25). In all cases the mean gaze position was not different from zero, as indicated by their 95%CI’s (data not shown), indicating that the participants generally looked straight ahead.

Next, we tested the look-ahead distance (Fig. 3). Values were log_e_-transformed to achieve normal distribution. The results of the distance model revealed a significant main effect for the shoe type (F_1,284_ = 6.4, *p* = 0.01) but not for the task type (F_1,284_ = 3.5, *p* = 0.06). Their interaction was also non-significant (F_1,284_ = 0.38, p = 0.54). The main effect of the shoe type indicated a mean difference of 0.1 ± 0.04SE (*p* = 0.01) between the shoe (9.2 ± 0.13SE) and the RS (9.1 ± 0.13SE) conditions. The trend noted for the task type indicated that during the dual-task condition, participants tended to look further away (mean difference 0.074 ± 0.039SE).

For a more in-depth analysis, we divided the look-ahead distance into three categories (as described in the Methods section) and tested the effect of the shoe- and task-type on the time spent looking within each category. The values of all categories were log_e_-transformed to achieve normal distribution.

**Figure 4:**
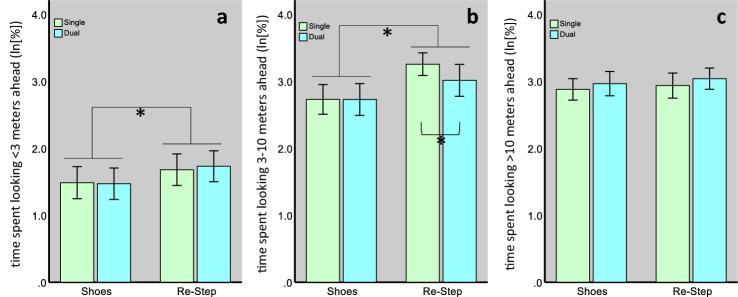
Percentage of time spent looking within the Short (**a**), Mid (**b**), and Long (**c**) distance categories by shoe type and task type. For both the Short and Mid distance categories, we found a significant main effect for the shoe type. For the Mid distance category, we also found a significant difference between single- and dual-task walks made with the RS. [*] indicates a significant difference. Bars represent mean values and error bars the 95% CI of the mean.

The model for the Short distance category (see Fig. 4) revealed only a significant effect for the shoe type (F_1,284_ = 10.3, *p* = 0.001), indicating that participants looked within this distance range for a greater percentage of time (MD 0.23 ± 0.07SE, *p* = 0.001), during the RS condition (1.71 ± 0.25SE) than during the shoe condition (1.48 ± 0.25SE). For the Mid distance category (see Fig. 4), we also found a significant main effect for the shoe type (F_1,284_ = 35.7, *p* < 0.001), and trends were noted for the task type (F_1,284_ = 3.2, *p* = 0.08) and for their interaction (F_1,284_ = 3.1, *p* = 0.08). These results indicate that participants looked within this range a greater percentage of the time, while walking with the RS system than with their own shoes (MD 0.41 ± 0.07SE, *p* = 6.96 × 10^–9^). Although the interaction term was non-significant, post-hoc pair-wise comparisons revealed that the effect of the shoe-type during the single-task walks (MD 0.53 ± 0.1SE, *p* = 9.81 × 10^–8^) was almost twice the magnitude of this effect during dual-task walks (MD 0.27 ± 0.1SE, *p* = 0.003). This effect was the consequence of a significant effect of the cognitive task on gaze behavior during RS walks (MD 0.24 ± 0.1SE, *p* = 0.01), but there was no change during walks made with every-day shoes (MD 0.001 ± 0.1SE, *p* = 0.99), indicating a cognitive-visual interference only during RS walks. As for the Long distance category, no effects were found for the percentage of time spent looking within this range (*p* > 0.18). As can be seen in Fig. 4, our conditions affected mainly the Mid distance category. Therefore, we calculated the median gaze distance within this category and found it to be 6.3 m. This gaze distance was unaffected be neither the shoe- (F_1,284_ = 0.56, *p* = 0.45) nor the task type (F_1,284_ = 0.62, *p* = 0.43).

**Figure 5:**
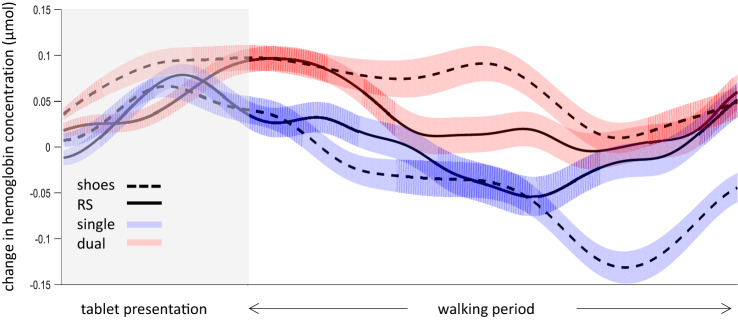
change in [HBO] over time under the different conditions. The shaded area represents the time at which the tablet was displayed, followed by the walking period (normalized to overcome differences in walking durations). Solid lines represent the mean response for the RS condition and dashed lines  for the shoes condition. Blue color represents the single task condition, and red  the dual task condition. *Due to the normalization procedure, some temporal shifting might have occurred.

*PFC activity*- The mean hemodynamic response functions (for HBO) in the different conditions are presented in Fig. 5, and the results of the statistical model are presented in Fig. 3. The model for HBO included “Side” as an additional *fixed* effect, to test for any differences between hemispheres, as we had previously observed^37^. The results of this model revealed a significant main effect for the task type (F_1,1723_ = 27.01, *p* < 0.001) and side (F_1,1723_ = 15.84, *p* < 0.001), but not for the shoe type (F_1,1723_ = 1.49, *p* = 0.22). A significant interaction between shoe and task type was also observed (F_1,1723_ = 5.30, *p* = 0.02). These significant main effects indicated a mean difference of 0.061 µmol ± 0.012SE (*p* = 2.17 × 10^–7^) between the dual- and single-task conditions, and a difference of 0.047 µmol ± 0.012SE (*p* = 7.16 × 10^–5^) between the right and left hemispheres. To explore the interaction term, we performed a post-hoc pairwise comparison. These comparisons revealed a mean difference of − 0.041 µmol ± 0.017SE (*p* = 0.01) between the shoes and RS during the single-task condition, but in the dual-task condition we found a mean difference between shoe types of 0.013 µmol ± 0.017SE, which was not statistically significant (*p* = 0.45). Moreover, for both shoe types we found a significant increase in Δ[HBO] during the dual-task condition compared to the single-task condition, but this increase was much larger in the shoe condition (0.088 µmol ± 0.017SE, *p* = 1.25 × 10^–7^) than in the RS condition (0.034 µmol ± 0.017SE, *p* = 0.04).

In the model for HHB we excluded the term “Side”, since it was not significant. This model revealed a significant main effect for neither the shoe type (F_1,1723_ = 0.98, *p* = 0.32) nor for the task type (F_1,1723_ = 1.02, *p* = 0.31), and their interaction was also non-significant (F_1,1723_ = 0.37, *p* = 0.54).

*Cognitive task*- The results of the models for the cognitive task revealed a main effect of the shoe type on the order recall (F_1,142_ = 4.1, *p* = 0.04), but not the digit recall (F_1,142_ = 0.00, *p* = 1). For the order recall, we found that participants erred in 21 walks while walking with the RS and in 11 walks with their shoes (out of 144 walks), making them twice (95%CI 1.02–5.54) as likely to err while walking with the RS.

Finally, we assessed the correlation between gaze behavior and PFC activity. We excluded the dual-task condition from this analysis since we were interested in the cognitive demands associated with instability. For this assessment, we built models that included PFC activity (change in [HBO]) as the dependent variable and shoe type as a *fixed* effect. Within this basic model, we added each gaze variable affected by the shoe type as a *fixed* effect, and the interaction between the shoe type and the gaze variable.

PFC activity and gaze behavior were found to be correlated. Specifically, for the parameter ‘OnPath percentage of time’, we found a negative correlation between gaze behavior and PFC activity (F_1,861_ = 21.65, *p* < 0.001, β =  − 0.003)—the greater the percentage of time spent looking onto the walking surface, the less PFC activity there was. For the parameter ‘vertical gaze’ position, a positive correlation was found (F_1,861_ = 8.92, *p* = 0.003, β = 0.003), indicating that gazing closer to the vanishing point was associated greater PFC activity (note that this was a positive correlation because we inversed the values of this parameter). Finally, the percentage of time spent looking within the Mid-distance category was also negatively correlated with PFC activity (F_1,860_ = 12.96, *p* < 0.001, β = -0.002), indicating that an increase in time spent looking within this distance range was associated with a decrease in PFC activity. For this last parameter (percentage spent looking within the Mid-distance category), we also found a significant interaction with the shoe type (F_1,860_ = 4.15, *p* = 0.04, β_(Shoes)_ =  − 0.004): the slope (i.e., coefficient) for walks made with every-day shoes was twice the magnitude of the slope observed for walks made with the RS.

## Discussion

In this study, we wanted to investigate whether gazing onto the walking surface is susceptible to cognitive interference and whether it is associated with PFC activity, shedding light on the mechanism through which visual information is used to resolve a unique instability problem.

To this end, we employed the Re-Step (RS) system. The effect of the RS system on gaze behavior is elsewhere discussed^35^ . Briefly, walking with the RS increased the tendency to gaze down onto the walking surface. We believe that all observed changes in gaze behavior are but consequences of participants’ spending more time looking within the Mid-range distance category. Thus, the primary effect of the RS was an increase in time spent looking roughly 6 m ahead (the Mid-range distance category), a distance much greater than the distances observed when visual information is used to modulate stepping (e.g., precise stepping, as on a staircase)^15,16,41^. This observation, along with the fact that perturbations were visually unpredictable, provide the first line of evidence that the tendency to gaze onto the walking surface is unlikely indicative of an attempt to use visual information to plan and modify subsequent steps, i.e., for anticipatory stepping control. So, why did the unique walking condition increase the propensity to gaze down onto the walking surface? Although there are several possibilities, we believe that gazing down changes the visual flow perceived by the observer. Given that this flow is important for postural control^27–30^, this change can make the flow more appropriate for feedback postural control^34^, which can alleviate the instability^16,17,42^.

Nevertheless, while this conclusion might be logical, participants also increased the time spent looking up to three meters ahead (the Short-range distance category), which is within the range of look-ahead distances reported when visual information is used to modulate stepping. Furthermore, even the increase in time spent looking within the Mid-range distance category can indicate that participants were trying to control subsequent steps using visual information. Specifically, the peripheral visual field, especially the lower visual field, in addition to the central visual field, is used for anticipatory control of stepping (reviewed in^42^). Thus, this change may indicate that participants were trying to attend (covertly) to their own legs/feet and/or the near walking surface. Overall, we cannot rule out with certainty the possibility that the observed change in gaze behavior represents habitual gaze behavior. That is, participants may have gazed down in an attempt to resolve the instability through conscious control of stepping simply out of habit, despite its futileness under the unique walking conditions. In an attempt to resolve this uncertainty, we explored the cognitive demands of walking in these conditions, and in particular the association between these demands and gaze behavior.

When studying the cognitive demands of posture and gait, interference methodology is most often used^24,43^. Another approach entails neuroimaging of cortical areas associated with cognitive resources, such as the PFC^4–6^. In this study, we employed both approaches. For interference in the cognitive domain, we found an increased error rate during walks made with the RS. Such interference suggests that the stability problem increases the demand for cognitive resources, leaving fewer resources available to perform the cognitive task. This observation is in line with previous reports (e.g.^26^) of an increase in cognitive demands associated with increased postural challenge. Nevertheless, this effect would be expected no matter what function(s) the downward gaze serves, because the instability itself was expected to make participants shift to a more conscious state of control. Thus, by itself this observation does not indicate whether participants gazed down to control stepping or to enhance postural control.

Using neuroimaging, we found an increase in PFC activity during single-task walks with the RS, compared to the walks in every-day shoes (and in dual-task walks compared to single-task walks). This observation is also in line with previous reports linking the PFC with the control of standing and walking (reviewed in^6,25^) and confirms previous observations from our laboratory^37^ in which PFC activity was shown to increase when participants walked with the RS in comparison to walking with their own shoes. However, much like the results for the cognitive task, such an increase would be expected in any case. In fact, both the increased cognitive cost and PFC activity indicate that while walking with the RS, participants shifted to a more conscious state of control^2^.

Yet, in both cases the type of association with gaze behavior may shed some light on the mechanism through which participants were utilizing visual information. A limited number of reports indicate that gaze behavior associated with conscious stepping control is susceptible to cognitive interference^19–23^. Among the relevant reports, an increase of roughly two-fold in the time spent looking at task-irrelevant areas was observed under cognitive load^20,21^, possibly indicating that participants are trying to disengage from task-relevant visual information^20^. While the true nature of this phenomenon is unclear, deciding where and when to look^44^, retaining visuospatial maps of the walking surface^20^, and/or planning future actions^19^ are all cognitive functions likely to be involved in conscious, visually aided stepping control. Using visual information for postural control is involuntary^27^ and does not seem to increase the cognitive demands associated with maintaining an upright stance^32,45^; in fact, it has been suggested that the availability (and/or sufficiency) of this information promotes the automaticity of both standing^32^ and walking^11^. Therefore, we assumed that if gazing onto the walking surface was used to resolve the instability by altering the visual flow to better control posture, this gaze behavior would be less susceptible to cognitive interference than if visual information was used for anticipatory stepping control.

In line with this assumption, the time spent looking onto the walking surface, the vertical gaze position, and the lookahead distance were all unaffected by the cognitive interference. Further, the time spent looking up to three meters ahead was also unaffected. This parameter is the one most likely to represent conscious control (i.e., habitual activity), given that when humans use visual information to modulate stepping, they most often look within this range of distances. On the other hand, the time spent looking 3–10 m ahead was affected, indicating some interference. Nevertheless, despite the reduction in time spent looking within this range, values were still significantly greater than those observed during walks made with every-day shoes (both in single- and dual-task conditions). Therefore, although most parameters were unaffected by cognitive loading, as expected, some interference was observed, which might be interpreted as an inconclusive finding with respect to our hypothesis.

That said, regardless of whether visual information is used to modulate stepping or control posture, one has to decide where and when to look in order to gather this information. This process has been described as a continuous sequence of decisions^44^ and is, thus, cognitively demanding. Therefore, some interference with gaze behavior dedicated to postural control is not totally unreasonable. Overall, we believe these results support our assumption insofar as downward gazing does not indicate a futile attempt to modulate stepping, albeit this evidence is not conclusive.

As for PFC activity, we performed a correlation analysis between parameters pertaining to downward gazing and PFC activity. Our assumption was that sensory input, if it provides information useful to performing the walking task, can alleviate the uncertainty and thereby reduce the cognitive demands of the task and PFC activity. This assumption is supported by several reports (reviewed in^2^) indicating that the absence or inadequacy of sensory input, including visual, leads to greater cognitive load and PFC activity during walking, indicating reduced automaticity. In the current set-up, visual input could not have alleviated the instability if it had been used for anticipatory stepping control; however, if used for other functions, such as feedback postural control, visual input may indeed have served the purpose of enhancing stability. Further, conscious modulation of gait has been reported to increase PFC activity^8–12^. Therefore, we expected that, if visual information was used for anticipatory stepping control, gazing down longer would correspond to an increase in PFC activity, but that, if this information was used for postural control, a reduction in PFC activity would be observed. In other words, a positive correlation between the time spent gazing downward and PFC activity would indicate anticipatory stepping control, and a negative correlation would indicate postural control.

Indeed, several parameters representing downward gazing correlated with PFC activity, in a manner consistent with this assumption. Further, the percentage of time spent looking within the Mid-range distance category negatively correlated with PFC activity. This correlation also revealed a significant interaction with the shoe type. While previous work revealed that downward head motion does not affect the NIRS signal^46^, this interaction further indicates that the correlation we found was not just a consequence of a downward head inclination. All correlations indicated that the greater the tendency to gaze down, the greater the reduction in PFC activity, as would be expected if visual information helped to alleviate the instability. Interestingly, the time spent gazing up to three meters ahead was uncorrelated with PFC activity, possibly reflecting the low percentage of time spent looking at the nearby surface, the low variability of this parameter, and the fact that PFC activity represented the average of the entire walk.

## Conclusions

The results of the present investigation support our hypothesis that gazing down while walking can serve functions other than just anticipatory stepping control. Our results suggest that while compromised walking stability did increase the attentional demands and PFC activity associated with walking, it also increased the tendency to gaze down, despite the futility of doing so as a mean for resolving the instability through anticipatory stepping control. In comparison to previous reports, this gaze behavior was only slightly affected by cognitive load, showing a small but significant interference effect. Further, the tendency to gaze down and PFC activity negatively correlate, indicating that downward gazing was associated with reduced PFC activity and that this gaze behavior therefore provided information helpful to resolve the instability.

## Limitations

While this study has several drawbacks, the most important one is the lack of appropriate control—specifically, a condition in which anticipatory control of stepping was required. Such a control condition would have enabled us to directly compare between the cognitive-visual interferences in the two states of control. Such a control condition would also enable us to investigate whether the direction in which downward gazing and PFC activity correlate will be inversed, as would follow from our hypothesis.

## Supplementary Information

Supplementary Information.

## Data Availability

The authors declare that the data supporting the findings of this study are available within the supplementary information files of this paper.

## References

[1] Armstrong DM (1988). The supraspinal control of mammalian locomotion. J. Physiol..

[2] Clark DJ (2015). Automaticity of walking: functional significance, mechanisms, measurement and rehabilitation strategies. Front. Hum. Neurosci..

[3] Yogev-Seligmann G, Hausdorff JM, Giladi N (2008). The role of executive function and attention in gait. Mov. Disord..

[4] Hamacher D, Herold F, Wiegel P, Hamacher D, Schega L (2015). Brain activity during walking: a systematic review. Neurosci. Biobehav. Rev..

[5] Vitorio R, Stuart S, Rochester L, Alcock L, Pantall A (2017). fNIRS response during walking - Artefact or cortical activity? A Systematic review. Neurosci. Biobehav. Rev..

[6] Herold F (2017). Functional near-infrared spectroscopy in movement science: a systematic review on cortical activity in postural and walking tasks. Neurophotonics.

[7] Miller EK, Cohen JD (2001). An integrative theory of prefrontal cortex function. Annu. Rev. Neurosci..

[8] Suzuki M (2004). Prefrontal and premotor cortices are involved in adapting walking and running speed on the treadmill: an optical imaging study. Neuroimage.

[9] Harada T, Miyai I, Suzuki M, Kubota K (2009). Gait capacity affects cortical activation patterns related to speed control in the elderly. Exp. Brain Res..

[10] Koenraadt KL, Roelofsen EG, Duysens J, Keijsers NL (2014). Cortical control of normal gait and precision stepping: an fNIRS study. Neuroimage.

[11] Clark DJ, Rose DK, Ring SA, Porges EC (2014). Utilization of central nervous system resources for preparation and performance of complex walking tasks in older adults. Front. Aging Neurosci..

[12] Mirelman A (2017). Effects of aging on prefrontal brain activation during challenging walking conditions. Brain Cogn..

[13] Patla AE (1997). Understanding the roles of vision in the control of human locomotion. Gait Posture.

[14] Patla AE, Vickers JN (1997). Where and when do we look as we approach and step over an obstacle in the travel path?. NeuroRep..

[15] Patla AE, Vickers JN (2003). How far ahead do we look when required to step on specific locations in the travel path during locomotion?. Exp. Brain Res..

[16] Matthis JS, Yates JL, Hayhoe MM (2018). Gaze and the control of foot placement when walking in natural terrain. Curr. Biol..

[17] Higuchi T (2013). Visuomotor control of human adaptive locomotion: understanding the anticipatory nature. Front. Psychol..

[18] Marigold DS, Patla AE (2008). Visual information from the lower visual field is important for walking across multi-surface terrain. Exp. Brain Res..

[19] Yamada M (2011). Fallers choose an early transfer gaze strategy during obstacle avoidance in dual-task condition. Aging Clin. Exp. Res..

[20] Ellmers TJ, Cocks AJ, Doumas M, Williams AM, Young WR (2016). Gazing into thin air: the dual-task costs of movement planning and execution during adaptive gait. PLoS ONE.

[21] Feld JA, Plummer P (2019). Visual scanning behavior during distracted walking in healthy young adults. Gait Posture.

[22] Miller AB, Lajoie K, Strath RA, Neima DR, Marigold DS (2018). Coordination of gaze behavior and foot placement during walking in persons with glaucoma. J. Glaucoma.

[23] Zukowski LA, Tennant JE, Iyigun G, Giuliani CA, Plummer P (2021). Dual-tasking impacts gait, cognitive performance, and gaze behavior during walking in a real-world environment in older adult fallers and non-fallers. Exp. Gerontol..

[24] Woollacott M, Shumway-Cook A (2002). Attention and the control of posture and gait: a review of an emerging area of research. Gait Posture.

[25] Wittenberg E, Thompson J, Nam CS, Franz JR (2017). Neuroimaging of human balance control: a systematic review. Front. Hum. Neurosci..

[26] Lajoie Y, Teasdale N, Bard C, Fleury M (1993). Attentional demands for static and dynamic equilibrium. Exp. Brain Res..

[27] Lee, D. N. & Lishman, J. Visual proprioceptive control of stance. Journal of human movement studies (1975).

[28] Stoffregen TA (1985). Flow structure versus retinal location in the optical control of stance. J. Exp. Psychol. Hum. Percept. Perform..

[29] Bardy BG, Warren WH, Kay BA (1996). Motion parallax is used to control postural sway during walking. Exp. Brain Res..

[30] Guerraz M, Sakellari V, Burchill P, Bronstein AM (2000). Influence of motion parallax in the control of spontaneous body sway. Exp. Brain Res..

[31] Warren WH, Kay BA, Yilmaz EH (1996). Visual control of posture during walking: functional specificity. J. Exp. Psychol. Hum. Percept. Perform..

[32] Stins J, Michielsen M, Roerdink M, Beek PJ (2009). Sway regularity reflects attentional involvement in postural control: Effects of expertise, vision and cognition. Gait Posture.

[33] Teo W, Goodwill AM, Hendy AM, Muthalib M, Macpherson H (2018). Sensory manipulation results in increased dorsolateral prefrontal cortex activation during static postural balance in sedentary older adults: An fNIRS study. Brain and behavior.

[34] Koren, Y. et al. Gazing down increases standing and walking postural steadiness. Royal Society Open Science 8, 201556 (2021). 10.1098/rsos.201556 PMC8074885 33959324

[35] Koren, Y. et al. Downward Gazing for Steadiness. preprint at https://www.biorxiv.org/content/10.1101/2020.02.28.969162v1 (2020).

[36] Ellmers TJ, Young WR (2018). Conscious motor control impairs attentional processing efficiency during precision stepping. Gait Posture.

[37] Koren Y, Parmet Y, Bar-Haim S (2019). Treading on the unknown increases prefrontal activity: A pilot fNIRS study. Gait Posture.

[38] Bar-Haim S, Harries N, Hutzler Y, Belokopytov M, Dobrov I (2013). Training to walk amid uncertainty with Re-Step: measurements and changes with perturbation training for hemiparesis and cerebral palsy. Disabil. Rehabil. Assist. Technol..

[39] Huppert TJ, Diamond SG, Franceschini MA, Boas DA (2009). HomER: a review of time-series analysis methods for near-infrared spectroscopy of the brain. Appl. Opt..

[40] Scholkmann F (2014). A review on continuous wave functional near-infrared spectroscopy and imaging instrumentation and methodology. Neuroimage.

[41] Marigold DS, Patla AE (2007). Gaze fixation patterns for negotiating complex ground terrain. Neuroscience.

[42] Marigold DS (2008). Role of peripheral visual cues in online visual guidance of locomotion. Exerc. Sport Sci. Rev..

[43] Al-Yahya E (2011). Cognitive motor interference while walking: a systematic review and meta-analysis. Neurosci. Biobehav. Rev..

[44] Hayhoe MM, Matthis JS (2018). Control of gaze in natural environments: effects of rewards and costs, uncertainty and memory in target selection. Interface Focus.

[45] Delafontaine A, Hansen C, Marolleau I, Kratzenstein S, Gouelle A (2020). Effect of a concurrent cognitive task, with stabilizing visual information and withdrawal, on body sway adaptation of parkinsonian’s patients in an off-medication state: a controlled study. Sensors.

[46] Iara de, A. I., Jörn M Horschig, Gerakaki, S., Wanrooij, M. M. v. & Willy, N J M Colier. Cerebral oxygenation responses to head movement measured with near-infrared spectroscopy Ser. 11638, March 05 2021).

