# Supplementary material for: Vision, cognition, and walking stability in young adults
Source: Sci Rep. 2022 Jan 11;12:513. doi: 10.1038/s41598-021-04540-w (PMC8752684; doi:10.1038/s41598-021-04540-w)
Supplement: Supplementary file 1 — Supplementary Information. [file 41598_2021_4540_MOESM1_ESM.docx]

**Vision, Cognition and Walking Stability**

Yogev Koren, Rotem Mairon, Ilay Sofer, Yisrael Parmet, Ohad Ben-Shahar, Simona Bar-Haim

In this experiment we monitored the gaze behavior and prefrontal activity of participants walking with their own shoes and with the Re-Step system (RS). For testing, participants were instructed to walk at a comfortable pace, in a well-lit hallway, along a 20-meter-long course with their own shoes and with the RS. To familiarize participants with the RS system, they walked the entire course six times before testing commenced. Participants performed 12 consecutive walks in each shoe type (i.e., two blocks consisting of 12 walks each). Before each walk, participants stood silently for 25 seconds, after which they were presented with a tablet displaying either a simple countdown from F to A or a random sequence of six digits, both at a 1 Hz frequency. Following the countdown/sequence, a “GO” cue was presented to indicate that the participant should start walking. Participants were instructed to memorize the random sequence, but to ignore the countdown. At the end of walks preceded with a random sequence, participants reported what they recalled, and their recollection was recorded. In our instructions, we emphasized that both recall of the digits and their order were important. The order of blocks and tasks within blocks were randomly assigned to each participant.

During the entire experiment, both gaze position and PFC activity were monitored. Gaze position was monitored using binocular eye-tracking glasses (ETG, SensoMotoric Instruments, Teltow, Germany), at a 60-Hz sampling rate. Before testing, the ETG was calibrated according to the manufacturer’s instructions, using a 3-point calibrating procedure. Using the software provided by the manufacturer (BeGaze, v.3.5), gaze positions were referenced to a scenery video, from a front-mounted camera. Each video was then manually screened, and calibration of the ETG, before each walk, was evaluated. If needed, post-hoc calibration was performed assuming participants fixated on the middle of the tablet at the time of initial presentation. Following this procedure, the scenery video and gaze position data were exported for further analysis.

To assess PFC activity, change in hemoglobin concentration was monitored using a wireless, continuous-wave fNIRS device (*PortaLight*, Artinis, The Netherlands). The device measures the absorption of near-infrared light by hemoglobin, in three channels (source-detector distances of 30, 35, and 40 millimeters) utilizing two wavelengths (760 and 850 nm). In this study, two devices were used (one for each hemisphere). They were positioned at 15% of the distance from nasion to inion (from nasion) and at 7% of the head circumference to the left and right from midline. This position was previously described ^1^ and demonstrated by MRI to roughly represent Brodmann’s area 10 ^2^. The probes were attached using a two-sided adhesive tape and covered with a black cloth to prevent ambient-light contamination. Sample frequency was set to 10 Hz and was continuous throughout the experiment. Raw intensities were collected using the software provided by the manufacturer (Oxysoft, version 3.0.53).

To process gaze data, following the procedure described above, the recorded video and raw gaze data coordinates were processed using a dedicated MATLAB (version 2016Rb) script. Before processing the data, each video was segmented into its individual trials, containing only the walking periods (i.e., from the time point of the “GO” cue until the end of the walk). For technical reasons, the boundaries of the corridor (i.e., intersection of the walls and floor) were marked, beforehand, using patterned tape. To process the videos, we initially extracted two image patches at the locations of these patterned tapes. The extraction was done manually by selecting a line segment over each marker tape in a single frame and extracting the patches at the midpoint of each line segment. The video was then processed automatically in a frame-by-frame manner.

In each frame, we used the manually extracted patches as templates to detect regions that contained the striped pattern of the marker tapes. To this end, we used a template-matching technique based on a normalized cross-correlation in the frequency domain between the patches and the frame. This process resulted in a grayscale map with higher values indicating regions that more likely belong to the marker tapes. By thresholding this map, we obtained a binary image indicating regions in the marker tapes.

Finally, we used the Hough transform to find the parameters of a straight line passing through these regions. Using these parameters, we calculated the point of intersection of these lines to represent the vanishing point. Raw gaze coordinates were then referenced to this point and the vertical distance between them (i.e., the difference between the Y-axis values in pixels) was calculated. These values are presented as percentage of the frame size. For sake of simplicity, we used the inverse of the vertical gaze–distance values such that large values indicate a gaze directed straight ahead (i.e., toward the horizon/vanishing point) and small values indicate a downward gaze. In the same way, we also calculated the horizontal gaze position (i.e., X-axis values), with negative values indicating a leftward gaze and positive values a rightward gaze. We also estimated the look-ahead distance based on the known corridor width (i.e., the distance between the marker tapes) and using perspective geometry calculations. This method allowed us to calculate gaze distance only when gaze position was directed between the patterned tapes, i.e., when gaze was directed onto the future path.

For preprocessing PFC hemoglobin concentration data, first, raw intensities were exported to MATLAB for pre-processing and converted to optical density. Motion artifacts were detected and corrected (see details below). Following artifact removal, quality of the signal recorded was assessed using several techniques: 1) a spectrum analysis of the whole time series was performed and was visually inspected to detect heart pulsation around 1-1.5 Hz. Detection of heart pulsation within the signal is indicative of a physiological measurement. 2) A signal-to-noise ratio (SNR) was calculated ^3^ for each channel separately. 3) Correlation analysis between channels was performed, and intra-optode correlations were evaluated [1]. Since there are no cut-off values for the above-mentioned methods, results were subjectively evaluated and suspected poor-quality channels were excluded from statistical analysis.

To process this data we used the *Homer2* (version 2.1), a MATLAB-based toolbox ^4^. The procedure was as follows: Raw intensity was converted to optical density (OD) using the *hmrIntensity2OD* function. Then, the *hmrMotionArtifactByChannel* function was used to identify motion artifacts, using a threshold of 10 standard deviations. These were corrected with the *hmrMotionCorrectSpline* function [3], after which, each data series was visually inspected. When needed, additional correction was performed using the *hmrMotionCorrectWavelet* function ^5^. These methods of artifact correction produced good results for large amplitudes and amplitudes similar to the physiological content of the NIRS signal ^6, 7^. Next, a band-pass filter with cutoff frequencies of 0.2 and 0.01 Hz was applied using the *hmrBandpassFilt* function. Finally, using the *hmrOD2Conc* function, OD was converted to relative concentrations after correcting for differential path-length factor (DPF) according to Scholkmann & Wolf ^8^.

Following these procedures, the walking period of each trial was normalized to 200 data points using spline interpolation to overcome differences in trials’ duration. Mean value was then calculated for each trial, hemisphere and channel to represent change in PFC activity. These values of oxy- and deoxy-hemoglobin were extracted from the time series and subtracted from the preceding rest period (i.e., the last 5 seconds of rest, just before the tablet was presented).

References

1. Mirelman, A. *et al*. Increased frontal brain activation during walking while dual tasking: an fNIRS study in healthy young adults. *Journal of neuroengineering and rehabilitation* **11**, 85 (2014).

2. Maidan, I. *et al*. Changes in oxygenated hemoglobin link freezing of gait to frontal activation in patients with Parkinson disease: an fNIRS study of transient motor-cognitive failures. *J. Neurol.* **262**, 899-908 (2015).

3. Xu, J. *et al*. FC-NIRS: A Functional Connectivity Analysis Tool for Near-Infrared Spectroscopy Data. *Biomed. Res. Int.* **2015**, 248724 (2015).

4. Huppert, T. J., Diamond, S. G., Franceschini, M. A. & Boas, D. A. HomER: a review of time-series analysis methods for near-infrared spectroscopy of the brain. *Appl. Opt.* **48**, D280-98 (2009).

5. Scholkmann, F., Spichtig, S., Muehlemann, T. & Wolf, M. How to detect and reduce movement artifacts in near-infrared imaging using moving standard deviation and spline interpolation. *Physiol. Meas.* **31**, 649-662 (2010).

6. Molavi, B. & Dumont, G. A. Wavelet-based motion artifact removal for functional near-infrared spectroscopy. *Physiol. Meas.* **33**, 259-270 (2012).

7. Cooper, R. J. *et al*. A systematic comparison of motion artifact correction techniques for functional near-infrared spectroscopy. *Front. Neurosci.* **6**, 147 (2012).

8. Scholkmann, F. & Wolf, M. General equation for the differential pathlength factor of the frontal human head depending on wavelength and age. *J. Biomed. Opt.* **18**, 105004 (2013).

stylefix
